# Association of Parental Oral Health Knowledge and Self-Efficacy with Early Childhood Caries and Oral Health Quality of Life in Texas Schoolchildren

**DOI:** 10.3390/ijerph22040513

**Published:** 2025-03-27

**Authors:** Shreela V. Sharma, Jeanette E. Deason, Mengxi Wang, Alejandra Garcia-Quintana, Ru-Jye Chuang, Kila Johnson, Shalisa Garner, Steven Kelder, Jose-Miguel Yamal

**Affiliations:** 1 Center for Health Equity, Department of Epidemiology, The University of Texas Health Science Center at Houston (UTHealth Houston) School of Public Health, Houston, TX 77030, USA; alejandra.garcia@uth.tmc.edu; 2 Department of Biostatistics and Data Science, Coordinating Center for Clinical Trials, The University of Texas Health Science Center at Houston (UTHealth Houston) School of Public Health, Houston, TX 77030, USA; jeanette.e.deason@uth.tmc.edu (J.E.D.); mengxi.wang@uth.tmc.edu (M.W.); jose-miguel.yamal@uth.tmc.edu (J.-M.Y.); 3 Center for Health Equity, Department of Health Promotion & Behavioral Sciences, The University of Texas Health Science Center at Houston (UTHealth Houston) School of Public Health, Houston, TX 77030, USA; ru-jye.chuang@uth.tmc.edu; 4 Community Health and Wellness Division, Dental Health and Prevention, Harris County Public Health, Houston, TX 77027, USA; kila.johnson@phs.hctx.net (K.J.); shalisa.garner@phs.hctx.net (S.G.); 5 Department of Health Promotion & Behavioral Sciences, Michael & Susan Dell Center for Healthy Living, The University of Texas Health Science Center at Austin (UTHealth Houston) School of Public Health, Houston, TX 78701, USA; steven.h.kelder@uth.tmc.edu

**Keywords:** oral health knowledge, oral health self-efficacy, child dental caries, oral health, oral health-related quality of life

## Abstract

Improving children’s oral health is a national priority. Parental knowledge and self-efficacy influence children’s health behaviors; however, the relationship between parental oral health-related knowledge, self-efficacy, child oral health-related quality of life (OHQoL), and dental caries remains unclear, especially among low-income, ethnically diverse U.S. families. This study assesses the relationship between parental oral health-related knowledge, self-efficacy, child dental caries, and OHQoL. This cross-sectional seco ndary analysis uses baseline data from a school-based cluster-randomized controlled trial among children (kindergarten–second grade). Dental assessments were conducted using the International Caries Detection and Assessment System (ICDAS) on site in schools at baseline (n = 34 schools, n = 1084 consenting parent–child dyads). Child OHQoL, parental knowledge, and self-efficacy were measured using validated self-report surveys. Associations between exposures and outcomes (child dental caries prevalence, child OHQoL) were evaluated by univariate and multivariable logistic and linear regressions, respectively. After adjusting for covariates, higher parental self-efficacy was associated with lower child OHQoL (lower score indicates higher quality of life) [beta = −0.16, 95% CI: −0.24, −0.09, *p* < 0.01] and lower odds of active dental caries [Adj OR: 0.95, 95% CI: 0.9, 0.99, *p* = 0.02]. No significant associations were noted for parental knowledge. These findings can inform future research, understanding how parental psychosocial factors influence dental caries prevention behaviors and risk, and inform interventions for children.

## 1. Introduction

Improving children’s oral health is a national priority per the U.S. Department of Health and Human Services Healthy People initiative. Healthy People 2030 has established fifteen oral health (OH) objectives to improve oral health and access to dental care for American children and adults [1]. Dental caries is one of the most prevalent chronic childhood diseases globally [2]. Preventing dental caries among children is achievable with lifestyle adjustments such as adequate oral hygiene, healthy nutrition, and access to care [3].

Dental disease significantly affects children and their families, and according to Oral Health in America: A Report from the Surgeon General in 2000 [4], over 51 million hours of school are missed annually due to dental-related problems. In Texas, the problem is quite severe. Data from the 2011–2016 National Health and Nutrition Examination Survey (NHANES) [5] indicate that ~55% of 6–8-year-old children in the United States (U.S.) have dental caries. In Texas, while the rates are similar in kindergarten, they increase to 68% of Texan third-grade children having dental caries in this age group [6]. The presence of dental caries can also impact the quality of life in children. Studies in diverse populations have consistently reported lower quality of life among children affected by dental caries [7,8,9,10,11]. Childhood is the ideal age to develop, ingrain, and promote oral health-enhancing behaviors (e.g., brushing, flossing, regular dental checkups, healthy eating) and to inhibit oral health-compromising behaviors (e.g., frequent consumption of sugary snacks and drinks) [12]. From the age of 6, the permanent dentition begins to replace the primary dentition. Newly erupted teeth are more susceptible to dental caries due to incomplete enamel maturation and occlusal anatomy [13]. Therefore, this age is considered critical for primary prevention through the promotion of a healthy lifestyle and oral health-related behaviors.

Young elementary school-aged children depend on their parents/caregivers to help manage their oral health-related behaviors [14,15]. Parental knowledge and self-efficacy are known to influence children’s health behaviors; however, the relationship between parental oral health knowledge and their self-efficacy and child oral health quality of life and caries prevalence remains unclear, especially among low-income, ethnically diverse U.S. families. The relationship between parental knowledge and self-efficacy related to child dental caries prevalence and oral health quality of life (OHQoL) among children in kindergarten grade from predominantly low-income, ethnically diverse families in Houston, Texas, is assessed in this study. It is hypothesized that lower child dental caries and improved OHQoL are associated with higher levels of parental knowledge and self-efficacy.

## 2. Materials and Methods

This is a cross-sectional secondary analysis of baseline data that were collected as part of the CATCH Healthy Smiles school-based cluster-randomized controlled trial among children in kindergarten through the end of grade 2 [16]. Because the unit of analysis is the school, recruitment was initiated by enrolling those from the list of eligible schools in the Greater Houston area. Recruitment and baseline measures were staggered across two school years (2021–2022 and 2022–2023) for ease of recruitment and measurement. All schools and study participants have the same follow-up time. As the trial is prospective, data will continue to be collected at subsequent time points to assess intervention outcomes.

### 2.1. Recruitment and Sample

Parents/guardians, children, and teachers from participating schools were invited to participate in this study. Details regarding the study design, recruitment, and baseline measures are provided elsewhere [16]. In brief, a letter of invitation to participate was sent home to parents/guardians via their children. Presentations were given by study staff to kindergarten-, first-, and second-grade teaching staff and parents/guardians at parent nights inviting them to participate in this research study. Parental consent to participate was collected electronically and using hard-copy paper-based consent packets sent home through the school. Written informed consent was obtained from all participating parents/guardians (for themselves and their child) and school staff. Regardless of whether a parent/guardian consented to their child participating in this study, all children in the participating schools and grades received the assigned curriculum as part of their usual school day. Only those parents consenting to this study and their children were measured.

#### 2.1.1. School Inclusion Criteria

The CATCH Healthy Smiles study setting consists of eligible schools that meet the following criteria: (1) the schools must be located in the Greater Houston, TX, metropolitan area, (2) more than 75% of the children must be enrolled in the free/reduced school lunch program, (3) children must have been enrolled in kindergarten for the 2021–2022 or 2022–2023 school year, (4) the assigned intervention program must be agreed to be implemented, and (5) participation in and assistance with the measurements must be agreed to.

#### 2.1.2. Parent–Child Dyad Inclusion Criteria

CATCH Healthy Smiles study inclusion criteria for the parent–child dyad: (1) signed and dated parent/guardian informed consent must be provided, (2) the same household adult must be willing to complete all the surveys for this study, (3) must be willing to maintain compliance with all study procedures, (4) the parent/guardian must have the ability to speak and read in English or Spanish at a 4th-grade level, (5) the child must be enrolled in the participating school in kindergarten grade for the 2021–2022 or 2022–2023 school year, with no existing family plans to move to a different school during the study period, and (6) the child must be able to participate in regular activities at school.

#### 2.1.3. Child Exclusion Criteria

Children who met either of the following criteria were excluded from participation in the CATCH Healthy Smiles study: (1) any condition/disorder that may make it difficult to conduct an accurate visual examination for dental caries (e.g., severe fluorosis, enamel hypoplasia, special dental setting needs, severe cleft palate) or (2) any condition or situation that may interfere with the child’s receipt of the curriculum components (e.g., a child consistently engaged with other therapies, instruction, or activities during the tooth brushing routine). Both exclusion criteria are listed on the consent forms for parents/guardians to help identify the need for exclusion. An exclusion criterion (e.g., severe fluorosis) may also be identified by dental examiners during the dental assessment. A total of 1084 children completed enrollment and baseline measures across 34 schools (n = 17 intervention; 17 comparison schools), constituting the final sample size for the analysis. The CATCH Healthy Smiles program [16,17] was implemented in schools in the intervention condition, while CATCH’s sun safety curriculum, Sunbeatables^®^ (CATCH Global Foundation, Austin, TX, USA) was offered by those in the control condition. The assigned curriculum was received by all students in the participating schools and grades, regardless of whether their parent/guardian opted to participate in this study (i.e., dental assessments, anthropometric measurements, surveys).

### 2.2. Study Measures

#### 2.2.1. Primary Outcomes

The primary outcomes of this study are child active dental caries lesions and child OHQoL. Dental assessments were conducted using the International Caries Detection and Assessment System (ICDAS) [18]. All assessments were conducted on site at each of the 34 schools at baseline. Dental assessment protocols and tools were designed with input from dentist investigators who have extensive experience working with pediatric public health dentistry. Visual examination of each tooth surface was conducted, in a designated area of the school, by trained dental examiners using the ICDAS method. To evaluate caries experience, lesion activity, and severity, missing and filled teeth were recorded as DFS/dfs and DFT/dft scores using a case report form. The ICDAS score was dichotomized for each child as having an active lesion with severity over 2 versus no active lesion.

OHQoL was measured through The Early Childhood Oral Health Impact Scale (ECOHIS) [19]. The ECOHIS is a 13-item parent-reported self-administered validated scale that measures dental pain, dental function, appearance, and family and social impact. OHQoL accounts for the social, emotional, and functional aspects of oral health. It incorporates measures like experiencing dental pain, dental self-image issues, and having trouble eating or pronouncing words due to dental issues. A summative score was computed from the 13 items, with a higher score indicating a lower quality of life and vice versa. The ECOHIS can also be assessed with two subscales for the child and the parent. The child ECOHIS includes 9 items; each item has a score range of 0–4, with a range of 0–36 for the total score. The parent ECOHIS includes 4 items; each item has a score range of 0–4, with a range of 0–16 for the total score.

#### 2.2.2. Child and Parent Sociodemographics

Child and parent sociodemographics at baseline were collected through parent self-report surveys ascertaining child and parent age, race/ethnicity, gender, socioeconomic status, education, language spoken at home, and family size. Surveys were bilingual in English and Spanish and sent home with the child through the school or electronically to the parent’s email or texted to their phone.

#### 2.2.3. Child Anthropometric Measurements

Child anthropometric measurements were taken at each of the 34 schools at baseline. A calibrated stadiometer and digital scale were used by trained study staff to measure the child participant’s height and weight, respectively. These data were used to compute child Body Mass Index (BMI) percentiles and determine child weight status using CDC guidelines [20].

#### 2.2.4. Exposure Variables

Parent oral health knowledge, self-efficacy, and child oral health behaviors were reported by parents. Parental knowledge of children’s oral health, including knowledge of baby teeth hygiene and tooth brushing, was measured using 6 items [21], and self-efficacy to care for their child’s oral health was measured using 5 items [22]. Response options were provided on a Likert scale, with mean scores computed for parental knowledge, ranging from 1 to 5. Each item on the parental self-efficacy measure has a score range of 0–4, with 0–20 for the total summative score, with higher scores indicating higher knowledge and higher self-efficacy. Child oral health behaviors measured for this study included tooth brushing and flossing frequency, dental visit frequency, and number of missed school days in the previous school year due to dental issues.

### 2.3. Statistical Analysis

The proposed exposure variables were parental knowledge and self-efficacy related to oral health, while the outcome variables were child dental caries prevalence and the child oral health-related quality of life score. The baseline demographics and oral health practices of participating children and families were summarized using counts and percentages for categorical variables and the mean and standard deviation (SD) or median and interquartile range for continuous variables. Groups were compared using Chi-square or Fisher’s exact tests for categorical variables and the Wilcoxon rank-sum test for continuous variables. Associations between exposures and the child dental caries prevalence outcome and child OHQoL outcome were evaluated by univariate and multivariable logistic regressions and linear regressions, respectively. Hosmer and Lemeshow purposeful selection was adopted to select variables for the multivariable models using likelihood ratio tests. Exposure variables were forced into the final models one at a time during the selection process regardless of significance levels in univariate analyses. Forest plots were created to visualize odds ratios and their 95% confidence intervals from the multivariable logistic regressions. All analyses were conducted using R version 4.3.2 (R Foundation for Statistical Computing, Vienna, Austria).

## 3. Results

At baseline, overall, participating children across the 34 study schools had a mean age of 5.5 years and were predominantly Hispanic (71%) or Black/African American (23%). Regarding child weight status, 18% were classified as overweight and another 20% were in the obese category. Fifty-one percent of the children had active dental caries, defined by an active lesion with a severity of more than 2, as scored on their teeth ICDAS assessment. The child mean OHQoL score was 2.6 (SD 3.84), indicating high child-related OHQoL, as higher scores indicate lower oral health-related quality of life. Almost half of the children were reported to brush once a day or less (48%), while 52% brushed more than once a day, and approximately 77% of the children were reported to have visited the dentist in the last year (Table 1).

On average, participating parents were predominantly female (88%), 33 years old, Hispanic (73%), and had a high school/GED level of education or less (69%). Parental OHQoL was, on average, 1.12 (SD: 2.04), indicating high OHQoL for the parent. Overall, parent oral health-related knowledge scores were relatively high (mean: 4.18, SD: 0.77, with a range of 0 to 5), while parent oral health-related self-efficacy scores were moderately high with a mean summative score of 14.8 (SD: 3.50). At the household level, most of the households had an annual income of <USD 35,000 per year (76%) and housed an average of five members of the family, and 81% reportedly received some form of government assistance (Table 1).

**Figure 1:**
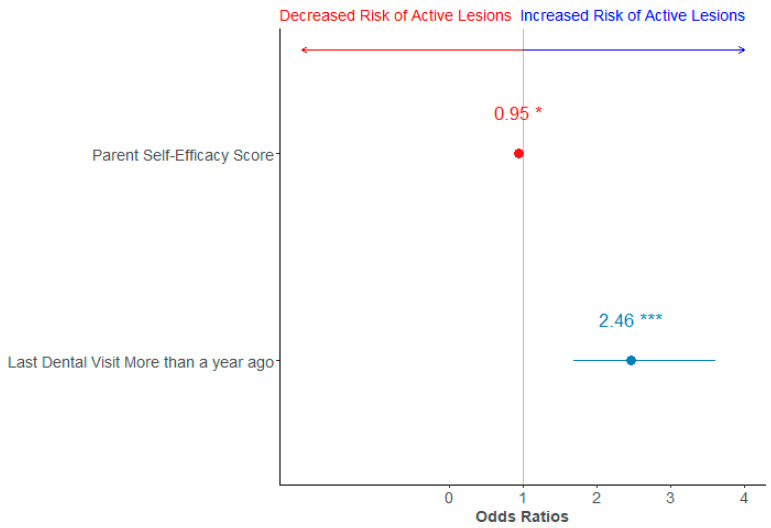
Forest plot of the adjusted logistic regression between active lesions and parental self-efficacy model. Variables associated with a decreased risk of active lesions are depicted in red, while those with an increased risk are in blue. * denotes *p*-value <0.05, *** denotes *p*-value <0.001.

In the regression analysis, parental oral knowledge was found to be significantly associated with child ECOHIS scores in the unadjusted model but was no longer associated after adjustment for the number of household members, the parental self-efficacy score, and child ethnicity. Higher parental self-efficacy scores were associated with lower child ECOHIS scores, both before and after adjustment (Table 2 and Figure 1). Of note, a lower ECOHIS score indicates a higher quality of life, while a higher score on the ECOHIS indicates lower oral health-related quality of life.

**Figure 2:**
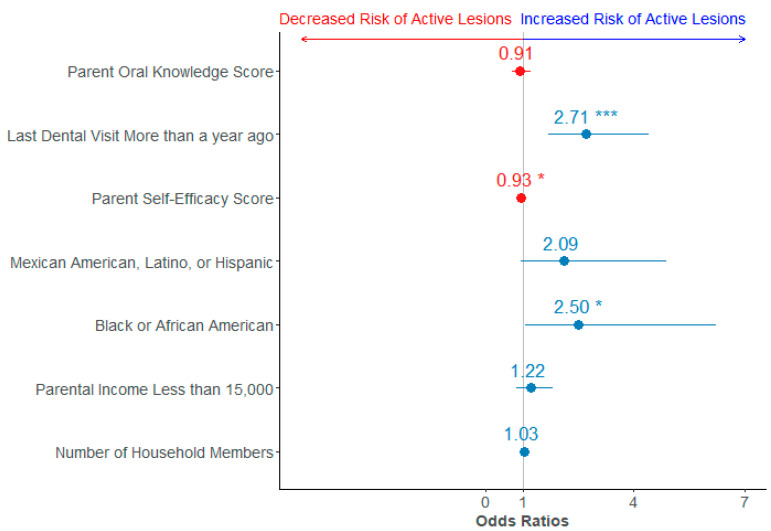
Forest plot of the adjusted logistic regression between active lesions and parental oral health knowledge; Variables associated with a decreased risk of active lesions are depicted in red, while those with an increased risk are in blue. * denotes *p*-value <0.05, *** denotes *p*-value <0.001.

Similarly, the parental oral health knowledge score was significantly associated with active dental caries lesions (OR = 0.8, 95% CI 0.66–0.98), but this was attenuated after adjustment. Higher parental self-efficacy scores were associated with lower odds of active dental caries lesions, with (8% lower) and without (5%) adjustment (Table 3 and Figure 2).

## 4. Discussion

Dental caries continues to persist at epidemic proportions in the U.S. among children [23]. To understand and prevent this pernicious health condition, it is important to assess the relationships between parental oral health-related psychosocial factors and child dental caries risk. The overall results of this study indicate that parental self-efficacy related to child oral health was significantly associated with child active dental caries prevalence and OHQoL, while no significant associations were observed for parental oral health-related knowledge. A significant negative association was found between parental self-efficacy and child active dental caries, indicating that higher parental confidence in addressing their child’s oral health needs was linked to lower caries prevalence. Regarding quality of life, parents with higher self-efficacy had significantly higher child OHQoL scores. Both these findings potentially indicate that improving parental oral health-related self-efficacy may be an important consideration in reducing dental caries risk and improving the related quality of life among their children.

Knowledge and self-efficacy are constructs from the Social Cognitive Theory (SCT) [24], a behavioral science theory, which is one of the underpinnings of our parent clinical trial [16]. The impact of a behavioral intervention grounded in SCT constructs on child incidence of dental caries and OHQoL is being assessed in our larger clinical trial. As such, the SCT posits that these psychosocial constructs interact with the environment to influence behavior. Parental knowledge, self-efficacy, and other psychosocial factors hypothesized to impact child oral health were measured in this study. Prior studies indicate that the findings are mixed regarding associations between parental psychosocial factors and child oral health outcomes, albeit parental self-efficacy has consistently been shown to be positively associated with child oral health, which concurs with our findings [21,22,25,26,27]. While some studies have demonstrated a significant positive relationship between parental knowledge and child oral health, other studies have shown mixed results, demonstrating a significant relationship between parental self-efficacy and child oral health but no associations with parental knowledge, as seen in our study [21,22,25]. Of note, prior studies have largely assessed child oral health-related behaviors (e.g., tooth brushing, oral hygiene practices, dental visits, diet) as the outcomes [21,22], and only one study has demonstrated a significant association between parental knowledge and child caries status among African American children [21]. Another study among Latino U.S. children reported that higher knowledge of dental utilization among mothers was associated with increased perceived susceptibility to early childhood caries in the child [28]. Broadly speaking, the hypothesized mechanism for these associations is that parental attitudes, knowledge, and beliefs may influence the choices parents make for their children, the behaviors they model to their children, and the tastes and preferences children develop throughout their childhood. These factors can, in turn, impact children’s dental caries risk [22]. However, there is a paucity of literature that has assessed these pathways, especially among diverse U.S. children. A cross-sectional study, using structural equation modeling among 651 children in grades 2 and 3 in China, reported a direct relationship between child oral health self-efficacy and oral health quality of life, albeit the relationship between knowledge and oral health quality of life was indirect through oral health self-efficacy and behaviors [29]. This may explain the findings seen in our study.

An additional explanation for our study findings may be that, while parental knowledge is important, it alone may not be sufficient to drive changes in oral health behaviors or outcomes [30]. Although knowledge can contribute to behavioral intentions, it may not be translated into action unless confidence in implementing that knowledge is also held by parents. The gap between knowledge and practice, often referred to as the “knowledge–behavior gap”, may help explain why a significant impact on children’s oral health outcomes was not observed for parental knowledge in this study [30]. Parental knowledge must likely be combined with other factors, such as self-efficacy and behavioral skills, for meaningful changes in child oral health to occur. Moreover, environmental factors and perceived barriers, particularly in lower-income families, may hinder the translation of knowledge into action, even when appropriate oral health practices are known by parents. The current body of literature on the associations between parent oral health-related knowledge and self-efficacy and child active dental caries prevalence and OHQoL is expanded by our study, which focuses on a large, predominantly low-income, ethnically diverse sample of kindergarten school-aged children in the U.S. Future efforts related to understanding the mechanisms by which parental psychosocial factors, such as self-efficacy, influence dental caries prevention behaviors, as well as the design and implementation of interventions for dental caries prevention in young U.S. children, can be informed by these findings.

The strengths of this study include a large sample size, the assessment of a diverse population, the use of validated assessment measures, and the objective measurement of child dental caries lesions by trained dentists [31] using ICDAS, which is considered a gold standard for dental caries assessment in research. Notwithstanding these strengths, limitations exist in this study. While the respondent gender distribution in this study was the same as in the ECOHIS validation study (predominantly female), differences were present in the race/ethnicity distribution and education level between the validation study and this study group. [19]. Although the ECOHIS has been validated in multiple languages and populations, it was not further validated in our study population, which represents a limitation. Another limitation includes missing responses on the parent surveys for the variables of interest. While extensive efforts were undertaken by the study team to reduce missingness in the responses, approximately 35% of the survey responses were missing. Furthermore, social desirability bias may bias survey responses. Finally, the cross-sectional nature of this study precludes causal inference due to a lack of temporality in the study design.

## 5. Conclusions

In conclusion, the results of our study demonstrate the following:

- A significant association was found between parental oral health self-efficacy for their child and child active dental caries prevalence and OHQoL.
- No associations were noted for parental oral health knowledge and child active dental caries prevalence and OHQoL.
- Future research is needed to understand the mechanisms by which parental psychosocial factors influence child dental caries-related behaviors and caries risk to design and inform theory-driven behavioral interventions for dental caries prevention in young children.

## Figures and Tables

**Table 1:** Baseline characteristics of study participants, overall and by active lesion status.

	Overall (N = 1084)	No Active Lesions ^a^ (N = 529)	Active Lesions ^a^ (N = 554)	*p* Value
Child age				
Mean (SD)	5.46 (0.554)	5.46 (0.536)	5.45 (0.570)	0.578
Median [Q1, Q3]	5.00 [5.00, 6.00]	5.00 [5.00, 6.00]	5.00 [5.00, 6.00]	
Child gender				
Female	546 (51.4%)	270 (52.5%)	276 (50.4%)	0.52
Male	517 (48.6%)	244 (47.5%)	272 (49.6%)	
Child ethnicity				
Black or African American	212 (22.6%)	110 (24.2%)	102 (21.2%)	0.358 ^b^
Mexican American, Latino, or Hispanic	663 (70.8%)	315 (69.4%)	347 (72.0%)	
White, Caucasian, or Anglo	42 (4.5%)	23 (5.1%)	19 (3.9%)	
Asian	7 (0.7%)	2 (0.4%)	5 (1.0%)	
Other	13 (1.4%)	4 (0.8%)	9 (1.9%)	
Parent age				
Mean (SD)	33.1 (7.75)	33.5 (7.63)	32.7 (7.86)	0.114
Median [Q1, Q3]	32.0 [28.0, 37.0]	32.0 [28.0, 38.0]	32.0 [27.0, 37.0]	
Parent gender				
Female	784 (88.3%)	390 (90.7%)	394 (86.0%)	0.039 *
Male	104 (11.7%)	40 (9.3%)	64 (14.0%)	
Parent ethnicity				
Black or African American	191 (21.8%)	95 (22.4%)	96 (21.1%)	0.009 ^b^
Mexican American, Latino, or Hispanic	638 (72.6%)	307 (72.4%)	330 (72.7%)	
White, Caucasian, or Anglo	29 (3.3%)	19 (4.5%)	10 (2.2%)	
Asian	9 (1.0%)	1 (0.2%)	8 (1.8%)	
Other	12 (1.4%)	2 (0.5%)	10 (2.2%)	
Language spoken at home				
Only English	284 (29.6%)	144 (30.7%)	140 (28.6%)	0.388
More English than another	125 (13.0%)	66 (14.1%)	59 (12.1%)	
Both English and another	148 (15.4%)	71 (15.1%)	77 (15.7%)	
More another than English	107 (11.2%)	43 (9.2%)	63 (12.9%)	
Only another	295 (30.8%)	145 (30.9%)	150 (30.7%)	
Parent education				
Never attended school or only attended kindergarten	47 (5.3%)	20 (4.6%)	27 (5.9%)	0.588
Grades 1 through 8 (elementary)	103 (11.5%)	52 (12.0%)	51 (11.1%)	
Grades 9 through 11 (some high school)	131 (14.7%)	59 (13.6%)	72 (15.7%)	
Grades 12 or GED (high school graduate)	337 (37.7%)	170 (39.3%)	167 (36.4%)	
College 1 year to 3 years (some college or technical school)	205 (23.0%)	94 (21.7%)	111 (24.2%)	
College 4 years or more (college graduate)	70 (7.7%)	38 (8.8%)	31 (6.8%)	
Parent employment status				
Employed for wages	351 (42.8%)	181 (45.6%)	170 (40.2%)	0.627 ^b^
Self-employed	56 (6.8%)	26 (6.5%)	30 (7.1%)	
Out of work for less than 1 year	30 (3.7%)	12 (3.0%)	18 (4.3%)	
Out of work for more than 1 year	45 (5.5%)	20 (5.0%)	25 (5.9%)	
Homemaker	274 (33.4%)	128 (32.2%)	145 (34.3%)	
Employed in seasonal labor	24 (2.9)	11 (2.8%)	13 (3.1%)	
Retired	3 (0.4%)	0 (0%)	3 (0.7%)	
Unable to work	38 (4.6%)	19 (4.8%)	19 (4.5%)	
Parent income				
Less than 10,000	176 (26.9%)	83 (25.6%)	92 (27.9%)	0.915
10,001 to 15,000	85 (13.0%)	39 (12.0%)	46 (13.9%)	
15,001 to 20,000	67 (10.2%)	35 (10.8%)	32 (9.7%)	
20,001 to 25,000	69 (10.5%)	37 (11.4%)	32 (9.7%)	
25,001 to 35,000	102 (15.6%)	49 (15.1%)	53 (16.1%)	
35,001 to 50,000	93 (14.2%)	47 (14.5%)	46 (13.9%)	
50,001 to 75,000 or greater	63 (9.6%)	34 (10.5%)	29 (8.8%)	
Household members				
Mean (SD)	4.89 (1.73)	4.88 (1.73)	4.91 (1.74)	0.832
Median [Q1, Q3]	5.00 [4.00, 6.00]	5.00 [4.00, 6.00]	5.00 [4.00, 6.00]	
Assistance programs				
No	166 (18.8%)	64 (15.0%)	102 (22.4%)	0.00624
Yes	719 (81.2%)	364 (85.0%)	354 (77.6%)	
Child BMI				
Underweight	30 (2.8%)	14 (2.7%)	16 (2.9%)	0.758
Healthy weight	633 (59.7%)	299 (58.3%)	334 (61.2%)	
Overweight	190 (17.9%)	97 (18.9%)	92 (16.8%)	
Obese	207 (19.5%)	103 (20.1%)	104 (19.0%)	
Child QOL (ECOHIS)				
Mean (SD)	2.61 (3.84)	1.97 (2.84)	3.24 (4.55)	<0.001 *
Median [Q1, Q3]	1.00 [0, 3.00]	1.00 [0, 2.00]	2.00 [0, 4.00]	
Parent QOL (ECOHIS)				
Mean (SD)	1.12 (2.04)	0.760 (1.46)	1.48 (2.43)	<0.001 *
Median [Q1, Q3]	0 [0, 2.00]	0 [0, 1.00]	0 [0, 2.00]	
Missed school days				
Mean (SD)	0.47 (1.16)	0.419 (0.989)	0.519 (1.30)	0.542
Median [Q1, Q3]	0 [0, 0]	0 [0, 0]	0 [0, 0]	
Child’s brushing frequency				
Never	6 (0.8%)	3 (0.8%)	3 (0.8%)	0.842 ^b^
Less than once a week	6 (0.8%)	2 (0.6%)	4 (1.1%)	
At least once a week but not everyday	51 (7.2%)	23 (6.4%)	28 (7.9%)	
Once a day	280 (39.3%)	140 (39.1%)	139 (39.4%)	
More than once a day	369 (51.8%)	190 (53.1%)	179 (50.7%)	
Child’s last dentist visit				
In the last year	541 (76.5%)	304 (84.7%)	236 (68.0%)	<0.001 *
More than 1 year ago but less than 2 years ago	67 (9.5%)	17 (4.7%)	50 (14.4%)	
More than 2 years ago	39 (5.5%)	15 (4.2%)	24 (6.9%)	
Never have been	60 (8.5%)	23 (6.4%)	37 (10.7%)	
Parental knowledge score				
Mean (SD)	4.18 (0.765)	4.24 (0.784)	4.12 (0.742)	0.00326 *
Median [Q1, Q3]	4.17 [3.83, 5.00]	4.33 [4.00, 5.00]	4.00 [3.67, 4.83]	
Parental self-efficacy score				
Mean (SD)	14.8 (3.50)	15.2 (3.41)	14.4 (3.55)	0.0029 *
Median [Q1, Q3]	15.0 [13.0, 17.0]	15.0 [13.0, 18.0]	15.0 [13.0, 17.0]	

^a^ Active lesion status could not be determined for one participant and is excluded from these columns. ^b^ *p*-values calculated using Fisher’s exact test, otherwise Wilcoxon rank-sum test or Pearson’s Chi-squared test for continuous or categorical variables, respectively. Missing values are excluded from the table, so counts may not add up to column total. * Significant value: *p* < 0.05.

**Table 2:** Linear regression results for the child quality of life (ECOHIS) outcome. The multivariable model was adjusted for variables selected using the Hosmer–Lemeshow method.

	Unadjusted		Adjusted	
	Estimate [95% CI]	*p* Value	Estimate [95% CI]	*p* Value
Parental oral knowledge score	−0.45 [−0.81, −0.08]	0.02 *	−0.19 [−0.6, 0.22] ^a^	0.37
Parental self-efficacy score	−0.21 [−0.29, −0.14]	<0.01 *	−0.16 [−0.24, −0.09] ^b^	<0.01 *

^a^ Adjusted for number of household members, parental self-efficacy score, and child ethnicity. ^b^ Adjusted for active lesions and missed school days due to dental issues. * Significant value: *p* < 0.05.

**Table 3:** Logistic regression odds ratio estimates for the active caries outcome based on ICDAS assessment. The multivariable model was adjusted for variables selected using the Hosmer–Lemeshow method.

	Unadjusted		Adjusted	
	Odds Ratio [95% CI]	*p* Value	Odds Ratio [95% CI]	*p* Value
Parental oral knowledge score	0.8 [0.66, 0.98]	0.03 *	0.91 [0.69, 1.2] ^a^	0.5
Parental self-efficacy score	0.93 [0.89, 0.98]	<0.01 *	0.95 [0.9, 0.99] ^b^	0.02 *

^a^ Adjusted for last dental visit, parental self-efficacy score, child ethnicity, parental annual income, and number of household members. b Adjusted for last dental visit > 1 year ago. * Significant value: *p* < 0.05.

## Data Availability

The datasets presented in this article are not readily available because the data are part of an ongoing study. However, the data will be made available after the primary outcome paper has been published. Requests for accessing the datasets should be directed to Shreela Sharma or Jose-Miguel Yamal.

## Abbreviations

The following abbreviations are used in this manuscript:

OHRQoL	Oral health-related quality of life
ICDAS	International Caries Detection and Assessment System
ECOHIS	Early Childhood Oral Health Impact Scale
DFS/dfs	Decayed and filled surfaces in permanent and primary teeth
DFT/dft	Decayed and filled permanent and primary teeth
SCT	Social Cognitive Theory

## References

[1] Centers for Disease Control and Prevention Oral Conditions–Healthy People 2030. https://odphp.health.gov/healthypeople/objectives-and-data/browse-objectives/oral-conditions.

[2] Huang G., Cao G., Liu J., Liu M. (2024). Global trends in incidence of caries in permanent teeth of children aged 5 through 14 years, 1990 through 2019. J. Am Dent. Assoc..

[3] Pitts N.B., Twetman S., Fisher J., Marsh P.D. (2021). Understanding Dental Caries as a Non-Communicable Disease. Br. Dent. J..

[4] US. Department of Health and Human Services (2000). Oral Health in America: A Report of the Surgeon General.

[5] Fleming E., Afful J. (2018). Prevalence of Total and Untreated Dental Caries Among Youth: United States, 2015–2016.

[6] Texas Department of State Health Services (2019). Dental Health Data and Reporting. Texas DSHS. https://www.dshs.texas.gov/dental-health/dental-health-data-reporting.

[7] Do L.G., Spencer A. (2007). Oral Health-Related Quality of Life of Children by Dental Caries and Fluorosis Experience. J. Public Health Dent..

[8] Wong H.M., McGrath C.P., King N.M., Lo E.C. (2011). Oral Health-Related Quality of Life in Hong Kong Preschool Children. Caries Res..

[9] Kramer P.F., Feldens C.A., Ferreira S.H., Bervian J., Rodrigues P.H., Peres M.A. (2013). Exploring the Impact of Oral Diseases and Disorders on Quality of Life of Preschool Children. Community Dent. Oral Epidemiol..

[10] Krisdapong S., Prasertsom P., Rattanarangsima K., Sheiham A. (2014). Associations Between Perceived Needs for Dental Treatment, Oral Health-Related Quality of Life, and Oral Diseases in School-Aged Thai Children. Community Dent. Oral Epidemiol..

[11] Ramos-Jorge J., Alencar B.M., Pordeus I.A., Soares M.E., Marques L.S., Ramos-Jorge M.L., Paiva S.M. (2015). Impact of Dental Caries on Quality of Life Among Preschool Children: Emphasis on the Type of Tooth and Stages of Progression. Eur. J. Oral Sci..

[12] Fleming P. (2015). Timetable for Oral Prevention in Childhood—A Current Opinion. Prog. Orthod..

[13] Lynch R.J. (2013). The Primary and Mixed Dentition, Post-Eruptive Enamel Maturation, and Dental Caries: A Review. Int. Dent. J..

[14] Pujar P., Subbareddy V.V. (2013). Evaluation of the Tooth Brushing Skills in Children Aged 6–12 Years. Eur. Arch. Paediatr. Dent..

[15] Krol D.M., Whelan K., The Section on Oral Health (2023). Maintaining and Improving the Oral Health of Young Children. Pediatrics.

[16] Chuang R.J., Byrd-Williams C., Yamal J.M., Johnson K., Kelder S., Nelson S., Mofleh D., Sharma S.V. (2022). Design for a Cluster Randomized Controlled Trial to Evaluate the Effects of the CATCH Healthy Smiles School-Based Oral Health Promotion Intervention Among Elementary School Children. Contemp. Clin. Trials Commun..

[17] Sharma S.V., Kelder S., Yamal J.M., Chuang R.J., Byrd-Williams C., Bona G., Bajaj N., Brito F., Neumann A.S. (2022). Development and Feasibility Testing of CATCH Healthy Smiles, an Oral Health Promotion Intervention for Prevention of Dental Caries Among Elementary School Children. J. Sch. Health.

[18] Ekstrand K.R., Gimenez T., Ferreira F.R., Mendes F.M., Braga M.M. (2018). The International Caries Detection and Assessment System–ICDAS: A Systematic Review. Caries Res..

[19] Pahel B.T., Rozier R.G., Slade G.D. (2007). Parental Perceptions of Children’s Oral Health: The Early Childhood Oral Health Impact Scale (ECOHIS). Health Qual. Life Outcomes.

[20] Centers for Disease Control and Prevention Child and Teen BMI Categories. BMI. https://www.cdc.gov/bmi/child-teen-calculator/bmi-categories.html.

[21] Finlayson T.L., Siefert K., Ismail A.I., Delva J., Sohn W. (2005). Reliability and Validity of Brief Measures of Oral Health-Related Knowledge, Fatalism, and Self-Efficacy in Mothers of African American Children. Pediatr. Dent..

[22] de Silva-Sanigorski A., Ashbolt R., Green J., Calache H., Keith B., Riggs E., Waters E. (2013). Parental Self-Efficacy and Oral Health-Related Knowledge Are Associated with Parent and Child Oral Health Behaviors and Self-Reported Oral Health Status. Community Dent. Oral Epidemiol..

[23] Centers for Disease Control and Prevention FastStats–Oral and Dental Health. https://www.cdc.gov/nchs/fastats/dental.htm.

[24] Bandura A. (2010). Social-Cognitive Theory.

[25] Hooley M., Skouteris H., Boganin C., Satur J., Kilpatrick N. (2012). Parental Influence and the Development of Dental Caries in Children Aged 0–6 Years: A Systematic Review of the literature. J. Dent..

[26] Smith S.R., Kroon J., Schwarzer R., Hamilton K. (2020). Parental Social-Cognitive Correlates of Preschoolers’ Oral Hygiene Behavior: A Systematic Review and Meta-Analysis. Soc. Sci. Med..

[27] Hevel D.J., Henshaw M., Endrighi R., Adams W.G., Heeren T., Jankowski A., Borrelli B. (2023). The Differential Predictive Utility of Two Caregiver-Targeted Self-Efficacy Measures to Promote Oral Health of Underserved Children. Health Psychol..

[28] Wilson A.R., Mulvahill M.J., Tiwari T. (2017). The Impact of Maternal Self-Efficacy and Oral Health Beliefs on Early Childhood Caries in Latino Children. Front Public Health..

[29] Zhao J., Shi H., Wang J., Huang R., Liu Y., Zhang Y., Jiang N., Wang T., Wang J., Xu X. (2022). Association of oral health knowledge, self-efficacy and behaviours with oral health-related quality of life in Chinese primary school children: A cross-sectional study. BMJ Open..

[30] Rimal R.N. (2000). Closing the Knowledge-Behavior Gap in Health Promotion: The Mediating Role of Self-Efficacy. Health Commun..

[31] Yamal J.M., Mofleh D., Chuang R.J., Wang M., Johnson K., Garcia-Quintana A., Titiloye T., Nelson S., Sharma S.V. (2024). Training Protocol and Calibration of the International Caries Detection and Assessment System in a School-Based Clinical Trial of Elementary School-Age Children. J. Public Health Dent..

